# Was bedeutet Systemrelevanz in Zeiten der Pandemie?

**DOI:** 10.1007/s11609-022-00464-y

**Published:** 2022-03-15

**Authors:** David Kaldewey

**Affiliations:** Forum Internationale Wissenschaft, Rheinische Friedrich-Wilhelms-Universität Bonn, Heussallee 18–24, 53113 Bonn, Deutschland

**Keywords:** Systemrelevanz, Finanzkrise, Coronakrise, Lockdown, Schulschließungen, Zeitdimension, Öffentliche Soziologie, Systemic relevance, Financial crisis, Corona crisis, Lockdown, School closures, Time dimension, Public sociology, Activité essentielle, Crise financière, Crise du coronavirus, Confinement, Fermeture d’écoles, Dimension temporelle, Sociologie publique

## Abstract

Der Beitrag geht der Frage nach, was Systemrelevanz in Zeiten der Pandemie bedeutet und inwieweit „Systemrelevanz“ über die Pandemie hinaus zu einem Baustein der gesellschaftspolitischen Semantik des 21. Jahrhunderts werden könnte. Zur Beantwortung dieser Frage werden empirische, theoretische und gedankenexperimentelle Überlegungen verknüpft. Der erste Teil versteht Systemrelevanz als Akteurskategorie und untersucht die Karriere und die Bedeutungsverschiebung des Begriffs in verschiedenen diskursiven Kontexten. Der zweite Teil versteht Systemrelevanz als analytische Kategorie und diskutiert drei theoretische Perspektiven, die einhergehen mit der Etablierung quasi-soziologischer, im Alltag und in der Politik verwendbarer Beobachtungsschemata. Der dritte Teil schließlich widmet sich den konzeptionellen Herausforderungen der Soziologie während und nach der Pandemie. Ausgehend von einer Leerstelle des Systemrelevanz-Diskurses wird die These entwickelt, dass wir über Systemrelevanz nicht sinnvoll sprechen können, ohne zugleich über die erwartete oder geplante Dauer zu sprechen, mit der in Krisensituationen bestimmte Institutionen außer Betrieb genommen oder in einen Minimalbetrieb versetzt werden.

## Abstract (en)

This article explores the question of what systemic relevance means in times of the pandemic and to what extent „systemic relevance“ could become a component of the socio-political semantics of the 21st century. To answer this question, empirical, theoretical, and thought-experimental considerations are being combined. The first part understands systemic relevance as an actor category and examines the term’s career and shifting meanings in different discursive contexts. The second part understands systemic relevance as an analytical category and discusses three theoretical perspectives that accompany the establishment of quasi-sociological observational schemes in everyday life and politics. Finally, the third part is devoted to the conceptual challenges for sociology during and after the pandemic. Starting from a conceptual gap in the discourse of systemic relevance, it is argued that we cannot talk meaningfully about systemic relevance without at the same time considering the expected or planned duration with which certain institutions are being closed down or put into minimal operation in critical situations.

## Résumé

Cet article s’interroge sur le sens de la notion d’ « activité essentielle » en temps de pandémie et sur sa capacité à devenir, au-delà de la pandémie, un élément constitutif de la sémantique de la politique sociétale au 21^e^ siècle. Pour répondre à cette question, l’article combine des réflexions d’ordre empirique, théorique et expérimental. La première partie conçoit la notion d’ « activité essentielle » comme une catégorie utilisée par les acteurs et analyse la carrière et le glissement de sens de cette notion dans divers contextes discursifs. La deuxième partie conçoit la notion d’activité essentielle comme une catégorie analytique et discute trois perspectives théoriques qui vont de pair avec l’établissement de grilles de lecture quasi-sociologiques utilisables dans la vie quotidienne et la politique. Enfin, la troisième partie est consacrée aux défis conceptuels rencontrés par la sociologie pendant et après la pandémie. À partir du constat d’un vide dans le discours sur la notion d’activité essentielle, la thèse développée ici est que nous ne pouvons pas parler d’activité essentielle de manière censée sans parler en même temps de la durée attendue ou prévue durant laquelle certaines institutions sont mises hors service ou en service minimum dans des situations de crise.

## Einleitung

Die Selbstverständlichkeit, mit der wir heute über Systemrelevanz sprechen, täuscht darüber hinweg, dass es sich um eine sehr junge wissenspolitische Kategorie handelt. Dieser spezifisch deutsche Begriff, für den es im Englischen kein Äquivalent gibt, findet in den 1960er-Jahren, im Zuge der allgemeinen Konjunktur des Systembegriffs, einen Weg in den semantischen Haushalt der Gesellschaft. Zu einer terminologischen Präzisierung oder Stabilisierung kommt es allerdings nicht. Erst die weltweite Finanzkrise in den Jahren 2007–2009 macht „Systemrelevanz“ zu einem zentralen Begriff des öffentlichen Diskurses mit relativ eng umrissener Bedeutung: Es geht um Finanz- und Kreditunternehmen, die derart mit der Gesamtwirtschaft verflochten sind, dass ihre Insolvenz systemgefährdend wäre für das globale Finanzsystem und damit indirekt für die komplexen, funktional differenzierten Gesellschaften der Gegenwart. Nachdem der Begriff mit dem Abflauen der Finanzkrise wieder etwas in den Hintergrund gerückt war, tauchte er im März 2020 sehr plötzlich und mit ganz anderen Konnotationen wieder auf. Mit der weltweiten Implementierung von Lockdowns zur Eindämmung der Corona-Pandemie lag die Frage auf dem Tisch, welche gesellschaftlichen Bereiche von den historisch noch nie dagewesenen Einschränkungen ausgenommen werden mussten. Hier drängte sich, zumal im deutschen Sprachraum, wiederum die Semantik der Systemrelevanz auf, und tatsächlich gewann der Begriff im öffentlich-politischen Diskurs innerhalb weniger Wochen eine ganz neue, umfassendere Bedeutung. Der vorliegende Essay geht vor diesem Hintergrund der Frage nach, was Systemrelevanz in Zeiten der Pandemie bedeutet und inwieweit man den Begriff über die Pandemie hinaus als einen Baustein der gesellschaftspolitischen Semantik des 21. Jahrhunderts begreifen kann.

Der Beitrag ist in zwei Hauptteile und einen Schlussteil gegliedert. Der erste Teil (Abschnitt 2) widmet sich der noch jungen Begriffsgeschichte, untersucht also die Karriere und die Bedeutungsverschiebung von „Systemrelevanz“ in verschiedenen Kontexten. Der Begriff wird hier als *Akteurskategorie* verstanden, es geht in deskriptiver Perspektive darum, wie in der Politik, in den Medien, in der Wissenschaft, aber auch im Alltag über Systemrelevanz gesprochen wird. Der zweite Teil (Abschnitt 3) dagegen argumentiert theoretisch und stellt die Frage, ob Systemrelevanz auch als *analytische Kategorie* präzisiert und damit als (sozial-)wissenschaftliches Beobachtungsschema eingesetzt werden könnte. Die Brisanz dieser Perspektive liegt darin, dass sich die Soziologie ausgehend von einem theoretisch fundierten Begriff der Systemrelevanz dazu äußern könnte, welche Bereiche der Gesellschaft aus welchen Gründen systemrelevant sind – und welche Konsequenzen ihre Schließung oder Pausierung über eine längere Zeit hätte. Gerade für eine öffentliche Soziologie – die im Zuge der Coronakrise in besonderer Weise gefordert ist (Dörre 2020; Rosa 2020) – geht es auch darum, den Unterschied zwischen Akteurskategorien und analytischen Kategorien transparent zu kommunizieren: Die demokratische Öffentlichkeit hat ein Interesse daran, zu verstehen, ob Rekurse auf Systemrelevanz in den Debatten zur Pandemie einfach eine politische Rhetorik darstellen oder ob damit auf ein robustes sozialwissenschaftliches Wissen rekurriert wird. Ausgehend von diesen Überlegungen erläutert der Schlussteil (Abschnitt 4) die empirischen und konzeptionellen Herausforderungen für die soziologische Forschung. Es wird auf eine Leerstelle des Systemrelevanz-Diskurses aufmerksam gemacht und die These entwickelt, dass wir über Systemrelevanz nicht sinnvoll sprechen können, ohne zugleich über die Dauer zu sprechen, mit der die Gesellschaft bestimmte Institutionen auf Eis legen kann, ohne dass diese nachhaltig Schaden nehmen.

## Systemrelevanz als Akteurskategorie

**Figure 1:**
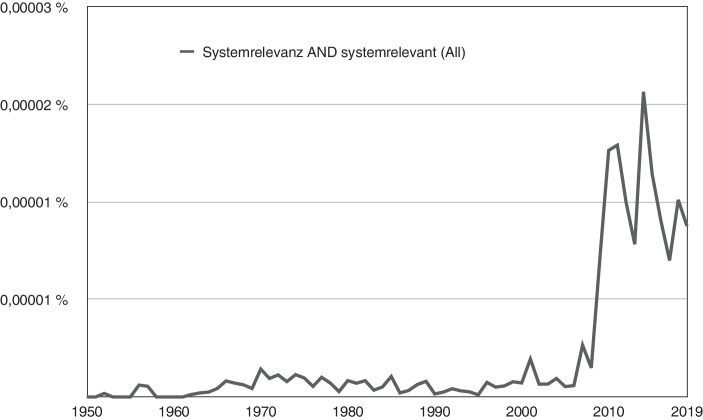

Für einen ersten Eindruck zur Entwicklung der Systemrelevanzsemantik bietet sich der Google Ngram Viewer an.^1^ Abbildung 1 zeigt, dass der Begriff der Systemrelevanz erst in den 1950er-Jahren sporadisch und ab Mitte der 1960er-Jahre – entsprechend der allgemeinen Konjunktur des Systembegriffs – etwas regelmäßiger auftaucht. Zwischen 2008 und 2010, also in Folge der Finanzkrise, verzehnfacht sich die relative Häufigkeit des Begriffs; womit ersichtlich ist, dass der Ausdruck in den folgenden Jahren faktisch für das „too-big-to-fail“-Prinzip wichtiger Finanzinstitutionen steht. Zwischen 2015 und 2019 lässt diese Begriffskonjunktur etwas nach, die weitere Entwicklung ist durch den Google Ngram Viewer nicht nachvollziehbar, da der Korpus nur Literatur bis 2019 enthält.

Im Blick auf die Begriffsentwicklung in der Pandemie gilt es deshalb, auf andere Medien und aktuellere Text-Korpora zurückzugreifen. Ein Medium, in dem solche Konjunkturen in sehr feiner Auflösung verfolgt werden können, ist Twitter. Während noch im Februar 2020 nur vereinzelte Tweets auf Systemrelevanz Bezug nehmen, explodieren diese im März 2020. Auffallend ist hier zunächst die Zunahme von Tweets, die auf die Systemrelevanz der Pflege verweisen – solche Tweets finden sich auch schon im Januar und Februar 2020, aber nun werden diese zunehmend mit der Corona-Pandemie verknüpft: „Jetzt wo alle sich einscheißen wegen #COVID19 ist Pflege plötzlich systemrelevant“ (@BoerneM, 3. März 2020). Dabei wird durchaus strategisch die Chance erkannt, in der gegebenen Situation die politischen Prioritäten zu verschieben: „Bauen wir gemeinsam an einem positiven #Framing für die #Systemrelevanz der #Pflege“ (@MarkusLauter, 16. März 2020). Die Erinnerung an die Finanzkrise ist noch spürbar, etwa wenn gefordert wird, angesichts der #carekrise „nicht nur Großunternehmen zu unterstützen“ (@katharinahoppe, 10. März 2020). Ein interessantes Fundstück hierbei ist der Tweet von @refnode (15. März 2020), der an einen Beitrag des Kabarettisten Volker Pispers über „Berufsgruppen, die diese Welt nicht braucht“ erinnert:

Machen Sie doch bitte folgendes Gedankenexperiment: Stellen Sie sich vor, morgen fallen alle Unternehmensberater, alle Investmentbanker und Aktienanalysten tot um, oder morgen fallen alle Krankenschwestern, alle Polizisten, alle Feuerwehrmänner und alle Altenpfleger tot um. Und überlegen Sie kurz, was Sie persönlich vermissen würden. (Pispers 2004)

Bemerkenswert ist, dass dieser Beitrag fünf Jahre vor der Finanzkrise und 15 Jahre vor der Coronakrise entstanden ist – der Systemrelevanzbegriff wird hier entsprechend noch nicht verwendet, aber in gewisser Weise in einer seiner neueren Bedeutungsdimensionen vorweggenommen. Das Beispiel zeigt auch, dass die Idee der Systemrelevanz unabhängig von der zunächst eher abstrakt-technischen Konnotation durchaus anschlussfähig ist im Kontext von Alltagskommunikation und Unterhaltung; es scheint sich um eine Semantik zu handeln, mit der man unmittelbar und ohne größere theoretische Fundierung arbeiten kann.

**Figure 2:**
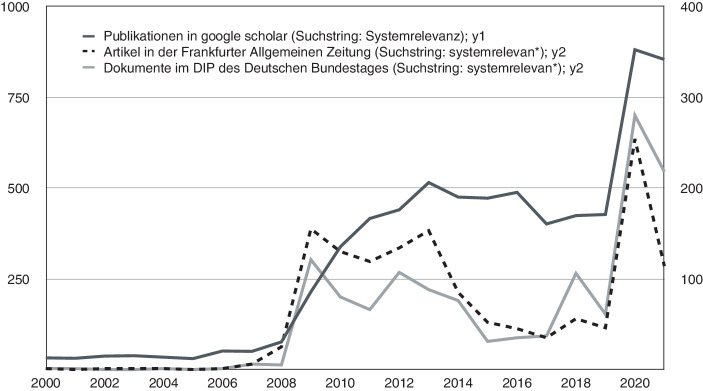

Um zu prüfen, ob die „zweite“ Karriere der Systemrelevanzsemantik bzw. deren Transposition von der Finanzkrise in die Coronakrise mehr ist als ein oberflächlicher Eindruck aus den sozialen Medien, muss die Begriffsverwendung in ausgewählten kommunikativen Kontexten etwas genauer angeschaut werden. Zu diesem Zweck werden im Folgenden drei verschiedene Korpora analysiert und die Häufigkeit von Dokumenten ausgezählt, in denen die Worte „Systemrelevanz“ oder „systemrelevant“ vorkommen. Der erste Korpus enthält bei Google Scholar indexierte wissenschaftliche Publikationen, der zweite Korpus Artikel aus der *FAZ* als Beispiel für eine überregionale Tageszeitung und der dritte Korpus Einträge im Dokumentations- und Informationssystem für Parlamentsmaterialien (DIP) des Deutschen Bundestages. Der erste Korpus steht, vereinfacht gesagt, für die Wissenschaft, der zweite für die Massenmedien, der dritte für die Politik. In Abbildung 2 sind diese Häufigkeiten im Zeitverlauf zwischen 2000 und 2021 abgebildet.^2^

Zunächst bestätigt diese Auswertung den ersten Eindruck des Google Ngram Viewers. In allen drei Korpora zeigt sich, dass der Begriff erst mit der Finanzkrise 2008 eine identifizierbare Rolle in der öffentlichen Kommunikation einnimmt. In der FAZ und in den DIP-Einträgen kann man sehen, dass diese Konjunktur etwa fünf Jahre anhält, bevor das Thema ab 2014 wieder an Relevanz verliert; während sich die Debatte in den wissenschaftlichen Publikationen länger hinzieht bzw. zeitverzögert erfolgt. Dann kommt das Jahr 2020 und die Zahlen verdoppeln bis verfünffachen sich. Wenn man nun in die konkreten Texte hineinliest, bestätigt sich, dass die frühere Bedeutung des Systemrelevanzbegriffs kaum noch eine Rolle spielt; zwar gibt es noch vereinzelte Bezüge, aber selbst diese werden innerhalb kurzer Zeit sehr viel seltener. Um diese Verschiebung zu illustrieren, listet Tabelle 1 alle Verwendungskontexte bzw. inhaltlichen Bezüge der 25 FAZ-Artikel auf, die den Begriff im Verlauf des März 2020 verwenden.

**Table 1:** 

07.03.2020	Hamsterkäufe („für unseren Stoffwechsel ist die Nudel eindeutig systemrelevant“)
10.03.2020	Systemrelevanz der Bank Lehman Brothers (nun im Kontext der Pandemie thematisiert)
11.03.2020	Sportphilosoph Gunter Gebauer über die Entbehrlichkeit von Fußball
14.03.2020	Systemrelevante Banken (ohne Bezug zur Pandemie)
16.03.2020	Schließung von Schulen und Kitas in allen Bundesländern
17.03.2020	Notbetreuung für die Kinder von Ärzten, Pflegern und Polizisten
18.03.2020	Systemrelevanz der öffentlich-rechtlichen Sender in der Pandemie
18.03.2020	Verkäufer in Supermärkten werden zu systemrelevanten Personen
19.03.2020	Maschinenbaukonzern wegen logistischer Ketten „indirekt systemrelevant“
20.03.2020	Option der Beschlagnahmung von systemrelevanten Gütern in Frankreich
20.03.2020	Rechtswissenschaftler Hinnerk Wißmann: Kritik an der Systemrelevanz-Unterscheidung
20.03.2020	LKW-Fahrer sind für die Aufrechterhaltung der Lieferketten systemrelevant
21.03.2020	Systemrelevanz des Journalismus
23.03.2020	Virologe Christian Drosten über die Verzichtbarkeit von Fußball
24.03.2020	Ungerechte Bezahlung von systemrelevanten Berufen in Pflege und Gesundheit
24.03.2020	Metareflexion: „Systemrelevant“ sind auf einmal nicht mehr Banken, sondern Berufe
24.03.2020	Olympia-Vorbereitung als systemrelevante Arbeit
25.03.2020	Systemrelevanz von Streifen-Polizisten (im Kontext einer Fernsehkritik)
25.03.2020	Systemrelevanz von Privatlaboren mit großen Testkapazitäten
26.03.2020	Systemrelevante Branchen auf dem Kapitalmarkt
26.03.2020	Eventim-Chef: Kultur ist kein Luxusgut, sondern systemrelevant
27.03.2020	Kommunen sind genauso systemrelevant wie die Wirtschaft
28.03.2020	Systemrelevante Berufe sind zugleich besonders riskante Berufe
28.03.2020	Systemrelevante Mitarbeiter bei den Feuerwehren und Rettungsdiensten
31.03.2020	„Systemrelevante PS“ (im Kontext einer Autokritik)

Nicht ohne Ironie ist, dass der Begriff mit Bezug auf die Pandemie das erste Mal am 7. März 2020 in einer Glosse auftaucht, die angesichts der anlaufenden Hamsterkäufe auf die bislang nicht registrierte Systemrelevanz von Nudeln hinweist. Am 10. und 14. März finden sich noch Artikel mit Bezug zu Banken bzw. zur Finanzkrise, ansonsten steht die Begriffsverwendung durchgehend im Zeichen der Pandemie. Dabei kommen auch Wissenschaftler^3^ zu Wort, etwa der Sportphilosoph Gunter Gebauer, der über die Entbehrlichkeit von Fußball schreibt und dabei erwähnt, dass Seuchenmediziner hier von „nichtvorhandener Systemrelevanz“ sprechen (11. März), oder der Virologe Christian Drosten, der sich ebenfalls zum Thema Fußball äußert: „Auf Dinge, die schön sind, aber nicht systemrelevant, wird man lange verzichten“ (23. März). Im Kontrast zu solchen Aussagen gibt sich der Rechtswissenschaftler Hinnerk Wißmann irritiert über die Vorstellung, „man könne in unserer ausdifferenzierten Gesellschaft auf längere Zeit ‚systemrelevante‘ von sonstigen Tätigkeiten unterscheiden“ (20. März). Genau diese Problematik findet sich – aus heutiger Sicht – zugespitzt in der Schlagzeile: „Nur noch das Systemrelevante: Als letztes Bundesland schließt Mecklenburg-Vorpommern Schulen und Kitas“ (16. März).^4^ In den letzten beiden Märzwochen wird dann immer wieder ausprobiert, welche sonstigen Bereiche und Rollen nach der Logik der Pandemie als systemrelevant gelten müssten oder zumindest in Betracht gezogen werden sollten, etwa Verkäufer in Supermärkten (18. März), LKW-Fahrer (20. März), der Journalismus (21. März), Berufe in Pflege und Gesundheit (24. März), Polizisten (25. März), die Kultur (26. März) oder die Kommunen (27. März). Dass man schließlich in einer Autokritik von „systemrelevanten PS“ liest (31. März), ist vielleicht kein Zufall, sondern zeigt an, wie schnell sich eine neue Vokabel im allgemeinen Sprachschatz wiederfindet.

Wie sieht es zur gleichen Zeit in der politischen Kommunikation aus? Wenn man hierzu die Bundestagsprotokolle heranzieht, zeigt sich, dass die Systemrelevanzsemantik im Zusammenhang mit der Corona-Pandemie zum ersten Mal in einer Debatte am 25. März 2020 auftaucht, in der es um diejenigen Menschen geht, die in der Pandemie besonderes leisten. Tabelle 2 enthält zur Illustration alle Zitate von Abgeordneten, die den Begriff an diesem Tag verwenden. Die Rede ist dabei von Beschäftigten im Gesundheitswesen und im Einzelhandel, Berufskraftfahrern, Polizistinnen, Krankenschwestern und Ärzten. Gefordert wird u.a. ein konkreter finanzieller Zuschlag bzw. ein Pflegebonus, zu dem es dann später auch gekommen ist. Unabhängig davon zeigt sich hier noch deutlicher als im FAZ-Korpus, dass eine Hauptlinie in der Bedeutungsverschiebung von „Systemrelevanz“ etwas mit der Auszeichnung bestimmter Berufe zu tun hat.

**Table 2:** 

**Amira Mohamed Ali** (DIE LINKE)	„Aber auch ich möchte zunächst auf die Menschen zu sprechen kommen, die in dieser Krise Herausragendes leisten. Das sind zum Beispiel die Beschäftigten im Gesundheitswesen, im Einzelhandel, die Berufskraftfahrer und viele andere in den sogenannten systemrelevanten Berufen. Ihnen wird in diesen Tagen zu Recht viel gedankt, auch hier in diesem Hause. (…) Ein Zuschlag von 500 Euro pro Monat für diejenigen, die in den systemrelevanten Berufen arbeiten, das ist das Mindeste.“
**Dr. Rolf Mützenich** (SPD)	„Deswegen will ich sagen: Ja, wir müssen zuerst all denen danken, die uns in dieser schweren Krise helfen: natürlich den Berufstätigen in den Gesundheits- und den Pflegeberufen, im Lebensmittelhandel, im Transportgewerbe. Und ja, sie werden heute als systemrelevant benannt. Aber ihr Lohn ist nicht so, wie wir ihn uns wünschen.“
**Alexander Dobrindt** (CDU/CSU)	„In Krisensituationen – wir haben ja in der Vergangenheit schon einige hinter uns gebracht – wird immer gerne über die Systemrelevanz gesprochen. Und gerade erleben wir jeden Tag, was Systemrelevanz in unserem ganzen Land vor Ort bedeutet. Es sind die Menschen in ihren Berufen, es sind die Krankenschwestern, die Ärzte, die Kräfte in den Supermärkten, die Polizistinnen und viele mehr. Meine Damen und Herren, wenn unser ganzes Land runterfährt, müssen diese Menschen richtig rauffahren.“
**Susanne Ferschl** (DIE LINKE)	„Nicht nur die Wirtschaft, auch die Menschen brauchen einen Rettungsschirm. (…) Und: Beschäftigte in systemrelevanten Berufen brauchen eine Gefahrenzulage in Höhe von 500 Euro.“

Da wissenschaftliche Publikationen nicht in Echtzeit publiziert werden, wäre es hinsichtlich des Google-Scholar-Korpus wenig sinnvoll, in den März 2020 hineinzuzoomen. Schon bei der Thematisierung der Systemrelevanzfrage im Kontext der Finanzkrise zeigt sich, dass dieser Korpus gewissermaßen träger, dafür aber nachhaltiger reagiert als die beiden anderen Korpora (Abbildung 2). Methodisch besteht außerdem das Problem, dass die meisten Publikationen nicht genau datiert, sondern nur mit einem Veröffentlichungsjahr versehen sind. Entsprechend bietet sich ein anderes Vorgehen an, nämlich ein Blick in die wissenschaftlichen Publikationen, die im zweiten Jahr der Pandemie erschienen sind. Ausgewählt wurden – am Tag der Recherche, dem 05.01.2022 – 342 deutschsprachige Publikationen, die Google Scholar auf 2021 datiert und die das Substantiv „Systemrelevanz“ enthalten. Von diesen Texten enthalten nur 25 % Wörter wie „Finanzkrise“, „Finanzwirtschaft“ oder „Banken“, womit sich bestätigt, dass der Begriff sich von diesem Ursprungskontext gelöst hat. Dagegen finden sich in 56 % dieser Publikationen Wörter wie „Gesundheitssystem“, „Krankenhaus“, „Pfleger“, „Pflegerinnen“, „Ärzte“ oder „Ärztinnen“; 59 % enthalten Wörter wie „Schule“, „Bildung“, „Lehrer“ oder „Lehrerinnen“. Weiter finden sich „Kunst“ oder „Kultur“ in 45 %, „Religion“ oder „Kirche“ in 28 % der Texte. Dazu kommen verschiedene Hinweise auf kritische Infrastrukturen (dazu unten mehr): Wörter wie „Infrastruktur“, „Lieferketten“, „Wasserversorgung“, „Energieversorgung“ oder „Kraftwerk“ tauchen in 42 % der Publikationen auf. Eine systematischere Inhaltsanalyse – anbieten würde sich etwa ein Topic Modeling – kann hier nicht geleistet werden, aber der kurze Einblick und das Querlesen dieser Publikationen lässt die Aussage zu, dass Systemrelevanz in der aktuellen wissenschaftlichen Literatur primär mit Berufen und sekundär mit gesellschaftlichen Funktionssystemen assoziiert ist. Dazu kommen in geringerem Maße – wobei sich das langfristig auch wieder ändern kann – Bezüge zur Finanzkrise sowie zu kritischen Infrastrukturen.

Zusammenfassend lässt sich festhalten, dass wir es mit einem Begriff der öffentlichen und politischen Sprache zu tun haben, der im 20. Jahrhundert noch fast keine Rolle spielt, im 21. Jahrhundert aber, ausgelöst durch zwei sehr unterschiedliche Krisen (Finanzkrise, Coronakrise) zu einer breit etablierten wissenspolitischen Kategorie geworden ist. Die aktuell dominierende Referenz des Begriffs sind bestimmte Berufe, die krisenbedingt ins Scheinwerferlicht geraten. Möglicherweise – das wird man erst in ein paar Jahren genauer einschätzen können – schreibt sich der Begriff der Systemrelevanz damit auch in die Alltagssprache ein. Zumindest fällt auf, dass er in vielen Kontexten als relativ theoriefreies und damit vielfältig anschlussfähiges Deutungsmuster fungiert. Man kann davon ausgehen, dass Menschen ihre eigene Tätigkeit und letztlich auch den eigenen Lebensentwurf in Zeiten gesellschaftlicher Krisen als „systemrelevant“ oder „nicht systemrelevant“ beobachten und damit ein Schema verwenden, das noch vor ein oder zwei Jahren nicht in gleicher Weise zur Verfügung stand. Von dieser Perspektive auf die alltägliche Verwendung von Systemrelevanz als *Akteurskategorie* unterschieden werden soll im nächsten Abschnitt eine stärker theoretisch-wissenschaftliche Perspektive. Gefragt wird, ob Systemrelevanz auch als *analytische Beobachterkategorie* funktioniert, und wenn ja, welche Theorien hier Vorarbeiten leisten.

## Systemrelevanz als analytische Kategorie

Die Frage, ob Systemrelevanz in bestimmten fachlichen Kontexten als wissenschaftlicher Begriff und damit als analytische Kategorie verwendet wird, muss zunächst mit Nein beantwortet werden. Zwar weckt der Begriff systemtheoretische Assoziationen und sein Auftauchen in der zweiten Hälfte des 20. Jahrhunderts korreliert mit der interdisziplinären Karriere des Systembegriffs, tatsächlich aber spielt er im systemtheoretischen Diskurs keine Rolle.^5^ Wenn im Folgenden drei theoretische Perspektiven unterschieden werden, dann ist „theoretisch“ nicht mit „wissenschaftlich“ gleichzusetzen, sondern meint jeweils einen in einem spezifischen diskursiven Kontext gebräuchlichen analytischen Zugang. Nur die erste, aus dem Kontext der Finanzkrise heraus entwickelte finanz- und rechtswissenschaftliche Perspektive läuft auf eine explizite Definition heraus, die aber der heute gängigen Begriffsverwendung in Politik, Medien und Wissenschaft nicht mehr gerecht wird. Um den jüngeren semantischen Verschiebungen gerecht zu werden, werden zwei weitere Perspektiven diskutiert, die zwar nicht explizit mit dem Begriff der Systemrelevanz arbeiten, aber in inhaltlicher Hinsicht eben dieses Problem adressieren: Die zweite Perspektive bezieht sich auf den Begriff der Kritischen Infrastrukturen (KRITIS), die dritte setzt bei der soziologischen Differenzierungstheorie an und greift dafür exemplarisch auf die konträren Positionierungen von Parsons und Luhmann zurück.

### „Too big to fail“

Eine terminologische Präzisierung hat der Begriff der Systemrelevanz erstmals, wie oben schon erwähnt, im Kontext der Finanzkrise und in der durch diese herausgeforderten Finanz-und Rechtspolitik erfahren. Für den vorliegenden Argumentationszusammenhang lohnt es aber nicht nur aus begriffshistorischen Gründen, an die Finanzkrise zu erinnern. Auch unabhängig vom Systemrelevanzdiskurs fällt auf, dass die Finanzkrise in der gesellschaftstheoretischen Reflexion der Coronakrise regelmäßig als Folie, mithin als Prototyp einer „Systemkrise“ (Lessenich 2020, S. 217) aufgerufen wird. Damit verbunden sind die zeitdiagnostischen Fragen nach der „Bestandsfähigkeit“ des demokratisch-kapitalistischen „Systems“ (Lessenich 2020, S. 2020) und der Notwendigkeit eines „Systemwechsels“ (Rosa 2020, S. 193). Während sich das Potenzial der vom späten Ulrich Beck avisierten „Metamorphose der Welt“ in der Finanzkrise noch nicht entfalten konnte, so vermutet Katharina Block in einem Essay aus der Frühphase der Pandemie, könnte die Moderne im Angesicht der Corona-Pandemie tatsächlich gezwungen sein, neue Wege einzuschlagen (Block 2020, S. 155 f.). Noch emphatischer verweist Hartmut Rosa darauf, dass in der neuen Krise ein handlungsfähiger Staat „die Interessen der Menschen und des Lebens gegen die Kapitalinteressen vertritt“ – und damit „Lebensrelevanz“ über „Systemrelevanz“ stelle (Rosa 2020, S. 202).^6^ Die folgenden Überlegungen sind zeitdiagnostisch zurückhaltender, können aber zumindest aufzeigen, dass und warum die Soziologie den Systemrelevanzbegriff nicht einfach den Finanz‑, Wirtschafts- und Rechtswissenschaften überlassen sollte.

Doch zunächst eine kurze Rückblende: Im Jahr 2009 schreibt der Rechtswissenschaftler Uwe H. Schneider, dass in der öffentlichen Diskussion weitgehende Einigkeit über die Notwendigkeit bestehe, den Zusammenbruch „systemisch relevanter Banken“ zu verhindern. Die Frage, was genau damit gemeint sei, beantwortet er mit drei Kriterien: Erstens gehe es um Institute, deren Insolvenz den Zusammenbruch weiterer Unternehmen im Finanzsektor auslösen würde („vertikaler Dominoeffekt“), zweitens um Institute, deren Insolvenz zu einem Zusammenbruch von Unternehmen der Realwirtschaft führe („horizontaler Dominoeffekt“), und drittens um solche, deren Insolvenz bei der Bevölkerung zu einem „Verlust des Systemvertrauens“ führen würde (Schneider 2009, S. 120; siehe auch Mülbert 2011). Es ist hier nicht der Ort, diese Definition und die vielfältigen sonstigen wissenschaftlichen Beiträge in diesem Bereich zu diskutieren und zu bewerten. Entscheidend ist, dass damals im globalen Zusammenspiel von staatlichen Akteuren und internationalen Organisationen der Begriff der Systemrelevanz seine Definition performativ gefunden hat – und es sich damit zuallererst um einen politischen Begriff handelt (Hübscher 2020, S. 126).^7^ In den USA und insgesamt im englischsprachigen Raum hat sich in diesem Zusammenhang das Schlagwort „too big to fail“ durchgesetzt, stilprägend war hier der gleichnamige Bestseller von Andrew Ross Sorkin (2009).

Schneider stellt in seinem Beitrag abschließend die Frage, ob es eigentlich plausibel ist, die vorgeschlagene dreidimensionale Definition von Systemrelevanz auf Kreditinstitute und Versicherungsunternehmen zu begrenzen, oder ob es auch andere Unternehmen geben könnte, „die wegen ihrer Größe, ihrer Schlüsselfunktion für die Wirtschaft, wegen des angesammelten Know-Hows oder wegen ihrer Bedeutung für die Daseinsvorsorge systemrelevant sind“. In dieser Frage klingt die Ahnung an, dass der im Rahmen der Finanzkrise relativ eng umrissene Begriff der Systemrelevanz auf eine viel umfassendere wirtschaftssoziologische Problemstellung verweist. Beantwortet wird die Frage nicht, wohl aber wird angedeutet, dass sie sich vielleicht schneller stellen könne, „als dem Autor lieb ist“ (Schneider 2009, S. 121). Ein Jahrzehnt später können wir einerseits festhalten, dass die Definition nicht auf weitere Unternehmen ausgeweitet wurde; andererseits können wir feststellen, dass der Fokus sich seit der Corona-Pandemie insgesamt weniger auf bestimmte Unternehmen, sondern eher auf Berufe und abstrakter gefasste gesellschaftliche Bereiche richtet. Damit liegt die Systemrelevanzfrage nicht mehr primär bei den Finanz‑, Wirtschafts- und Rechtswissenschaften, sondern bei den Sozialwissenschaften im Allgemeinen und nicht zuletzt bei der Soziologie.

Festgehalten werden kann weiter, dass der Systemrelevanzbegriff im Kontext des Finanzkrisendiskurses zwar analytisch einigermaßen scharf umrissen, zugleich aber stark politisiert ist. Der Begriff lässt sich einerseits als Akteurskategorie verstehen, da er von identifizierbaren Akteuren rhetorisch eingesetzt wurde, um ordnungspolitische Strategien zu plausibilisieren. Andererseits lässt er sich, da der politische Prozess wissenschaftlich begleitet wurde, auch als analytische Kategorie verstehen. Falsch wäre nur die Annahme, dass die Expertinnen vorweg eine Theorie mit scharfen Begriffen bereitgehalten hätten, die anschließend dann zur Krisenbearbeitung verwendet werden konnte. Treffender ist es, mit Sheila Jasanoff (2004) von einer Koproduktion von Wissenschaft und sozialer Ordnung auszugehen: Politisierte Akteurskategorien und wissenschaftlich-analytische Beobachterkategorien befruchten sich gegenseitig.

### Kritische Infrastrukturen

Wenn wir nun den Krisenkontext wieder wechseln und die im März 2020 einsetzenden sozialwissenschaftlichen Diskussionen über Systemrelevanz im Kontext der Corona-Pandemie in den Blick nehmen, dann fällt auf, dass Autorinnen und Autoren aus verschiedenen Disziplinen – etwa aus der Arbeitsmarktforschung (Burstedde et al. 2020, Lübker und Zucco 2020), den Gender Studies (Villa 2020), der Sozialen Arbeit (Wagner 2020) und der Wirtschaftsethik (Hübscher 2020) – Bezüge herstellen zum seit über zehn Jahren etablierten Begriff der „Kritischen Infrastrukturen“ (KRITIS). Auch bei diesem handelt es sich um einen primär politisch definierten Begriff, der zwar durch Erkenntnisse aus der Technikfolgenabschätzung und Risikoforschung beeinflusst sein dürfte, aber nicht im engeren Sinne als Fachbegriff innerhalb dieser Forschungsfelder gelten kann. Eingeführt wurde das Konzept vielmehr mit der von der Bundesregierung auf Initiative des damaligen Bundesinnenministers Wolfgang Schäuble hin beschlossenen „Nationalen Strategie zum Schutz Kritischer Infrastrukturen“ (BMI 2009). Die Bundesregierung, so heißt es in diesem Dokument, sieht in der Gewährleistung des Schutzes Kritischer Infrastrukturen bereits seit Ende der 1990er-Jahre eine „Kernaufgabe staatlicher Sicherheitsvorsorge“.^8^ Die offizielle und bis heute auf der gemeinsam vom Bundesamt für Bevölkerungsschutz und Katastrophenhilfe (BBK) und vom Bundesamt für Sicherheit in der Informationstechnik (BSI) betriebenen Website (www.kritis.bund.de) abrufbare Definition lautet wie folgt:

Kritische Infrastrukturen sind Organisationen und Einrichtungen mit wichtiger Bedeutung für das staatliche Gemeinwesen, bei deren Ausfall oder Beeinträchtigung nachhaltig wirkende Versorgungsengpässe, erhebliche Störungen der öffentlichen Sicherheit oder andere dramatische Folgen eintreten würden. (BMI 2009, S. 3)

Was die KRITIS-Strategie soziologisch interessant macht, ist eine Kategorisierung von für die Gesamtgesellschaft unverzichtbaren sozio-technischen Systemen nach dem Muster funktionalistischer Differenzierungstheorien. In einer vom BMI und den entsprechenden Behörden (BBK und BSI) bis Juli 2021 verwendeten vereinfachten Darstellung sind dies neun „Sektoren“, innerhalb derer dann jeweils nochmal spezifizierte „Branchen“ unterschieden werden (Tab. 3).^9^

**Table 3:** 

**Sektoren**	**Branchen**
Energie	Elektrizität Gas Mineralöl Fernwärme
Gesundheit	medizinische Versorgung Arzneimittel und Impfstoffe
Staat und Verwaltung	Regierung und Verwaltung Parlament Justizeinrichtungen Notfall‑/Rettungswesen einschl. Katastrophenschutz
Ernährung	Ernährungswirtschaft Lebensmittelhandel
Transport und Verkehr	Luftfahrt Seeschifffahrt Binnenschifffahrt Schienenverkehr Straßenverkehr Logistik
Finanz- und Versicherungswesen	Banken Börsen Versicherungen Finanzdienstleister
Informationstechnik und Telekommunikation	Telekommunikation Informationstechnik
Medien und Kultur	Rundfunk (Fernsehen und Radio) gedruckte und elektronische Presse Kulturgut symbolträchtige Bauwerke
Wasser	öffentliche Wasserversorgung öffentliche Abwasserbeseitigung

Eine zweite, daran anschließende und nun auch rechtlich kodifizierte Definition findet sich in der Verordnung zur Bestimmung Kritischer Infrastrukturen nach dem BSI-Gesetz (BSI-KritisV). Auffallend ist, dass in diesem Gesetz die Bereiche „Staat und Verwaltung“ sowie „Medien und Kultur“ nicht mehr aufgeführt werden, es wird also nur noch von sieben relevanten Sektoren ausgegangen.^10^ Als system- bzw. versorgungsrelevant gelten dann Organisationen und Anlagen aus diesen sieben Sektoren, deren Leistung im Gesetz genau bestimmte Schwellenwerte überschreitet, also bspw. Stromerzeugungsanlagen mit einer installierten Nennleistung von mehr als 420 MW oder Lebensmittelproduzenten, die pro Jahr mindestens 434.000 Tonnen Speisen oder 350 Mio. Liter Getränke prozessieren. Ohne hier weiter ins Detail zu gehen, kann festgehalten werden, dass es im Kern darauf ankommt, welche Großorganisationen im Katastrophenfall in Betrieb gehalten werden müssen. Das Spektrum der Gefahren, die zur Herausforderung für die Kritischen Infrastrukturen werden können, beinhaltet Naturereignisse, technisches/menschliches Versagen sowie Terrorismus, Kriminalität und Krieg (BMI 2009, S. 7). Auch an Epidemien und Pandemien wurde gedacht, sodass man die Coronakrise durchaus als einen Praxistest begreifen könnte, der offenlegt, ob und in welcher Weise die KRITIS-Strategie für die Pandemiepolitik und das Pandemiemanagement hilfreich war bzw. ist. Eine solche Bewertung würde den Rahmen des vorliegenden Beitrages allerdings sprengen; stattdessen konzentrieren sich die folgenden Anmerkungen auf Bedeutungsverschiebungen und Spannungen im diskursiven Schnittfeld von Systemrelevanz und kritischer Infrastruktur während der Frühphase der Pandemiebekämpfung.

Ausgehend von der damals (und bis heute) von Wissenschaft und Politik geteilten Annahme, dass die für den Verlauf der Pandemie entscheidende Variable die Anzahl der potenziell zu einer Ansteckung führenden sozialen Kontakte ist, setzte man in Deutschland ähnlich wie in weiten Teilen der Welt auf die Strategie des „Social Distancing“ – und zur Operationalisierung derselben auf einen mehr oder weniger weitgehenden „Lockdown“ des gesellschaftlichen Lebens (zur Radikalität dieses Vorgehens vgl. Caduff 2020). Der damit einhergehende Fokus auf jeden einzelnen Menschen als Träger des Virus und als potenzielles Opfer einer Infektion steht in auffallendem Kontrast zum abstrakt-technischen Duktus der KRITIS-Strategie. In dieser ging es, quasi-soziologisch und quasi-funktionalistisch, um *Institutionen*, die in Betrieb gehalten werden müssen. Nun aber, in der konkreten Krise, ging es darum, dass dieser Betrieb der Gesellschaft die *Menschen* weiter miteinander in Kontakt bringt und eben daraus eine Gefahr erwächst. Als Folge wurden also einerseits – mit der Logik der KRITIS-Strategie übereinstimmend – bestimmte Bereiche offengehalten, *obwohl* dort Menschen interagieren, während andererseits möglichst viele Bereiche geschlossen wurden, *weil* dort Menschen zusammenkommen.

Dazu kam ein eigentlich naheliegendes Problem, das in der KRITIS-Strategie aber nicht antizipiert worden war: Viele in systemrelevanten Bereichen tätige Mütter oder Väter waren darauf angewiesen, dass ihre Kinder in Kitas und Schulen betreut werden. Die historisch einmaligen flächendeckenden Kita- und Schulschließungen gingen deshalb einher mit dem Beschluss, einen „Notbetrieb“ für diejenigen Kinder aufrechtzuerhalten, deren Eltern in systemrelevanten Bereichen tätig sind. In der öffentlichen Ankündigung ging es dabei zunächst um Kinder von Krankenpflegerinnen und Ärztinnen, in der Praxis aber mussten die hier zuständigen Bundesländer innerhalb weniger Tage konkretisieren, welche Eltern das Privileg der Kinderbetreuung und Beschulung in Anspruch nehmen durften. In genau dieser Situation drängte sich der Begriff der Systemrelevanz auf und wurde gewissermaßen über Nacht zu einer wissenspolitischen Kategorie, die nicht mehr primär abstrakte Sektoren oder Infrastrukturen betraf, sondern konkrete Berufe und damit potenziell jedes berufstätige Individuum. Auffallend ist, dass die Bundesländer, auch wenn sie im Detail verschiedene Verordnungen erließen, zu diesem Zweck in erster Linie auf die in der KRITIS-Strategie definierten Sektoren und Branchen Bezug nahmen. Das BBK wiederum publizierte am 30. März 2020 unter dem Titel „‚Kritische Infrastruktur‘ in Vorgaben zur Notbetreuung in Schulen und Kitas“ einen Überblick zu den Regelungen der Länder und hat diesen seither mehrfach aktualisiert.^11^ Diese Verordnungen regierten unmittelbar in den Alltag vieler Familien hinein: „Für viele, die sich selbst für wichtig halten, waren die entsprechenden Verordnungen und Erlasse ernüchternd“ (Lübker und Zucco 2020, S. 472). Dazu kamen unterschiedliche Regelungen bezüglich der Frage, ob beide Elternteile in systemrelevanten Berufen arbeiten müssen, um Notbetreuung in Anspruch zu nehmen. Zumindest im ersten Lockdown – später wurden die Regelungen eher gelockert – konnte es beispielsweise sein, dass der Manager zuhause bleiben musste, damit seine Frau, die Krankenpflegerin, zur Arbeit gehen konnte.^12^

Neben dieser Verschiebung des Fokus von abstrakten Sektoren und Branchen zu konkreten Berufen und Menschen drängte sich vielen Beobachterinnen aus Politik, Wissenschaft und Medien schon früh auf, dass offensichtlich kritische Bereiche im KRITIS-Schema nicht abgebildet sind – insbesondere der Bereich der Pflege und der Sozialen Arbeit. In den Verordnungen der Länder wurde dies umgehend korrigiert, die Altenpflege etwa wurde durchgängig als systemrelevant definiert (vgl. Lübker und Zucco 2020, S. 474). Als Beispiel sei auf die vom nordrhein-westfälischen Ministerium für Arbeit, Gesundheit und Soziales am 15. März 2020 veröffentlichte „Leitlinie zur Bestimmung des Personals kritischer Infrastrukturen“ verwiesen (MAGS-NRW 2020). Man beachte zunächst, dass diese noch nicht explizit über „Systemrelevanz“ spricht, vielmehr übernimmt sie unmittelbar die in der KRITIS-Strategie vorgeschlagene Kategorisierung von neun Sektoren, ergänzt aber beim Sektor Gesundheit den Bereich der Pflege. Weiter wird ein „10. Sektor“ zur „Sicherstellung notwendiger Betreuung“ – sprich: Notbetreuung – eingeführt; genannt werden Schulen, Kindertageseinrichtungen, stationäre Einrichtungen der Kinder- und Jugendhilfe sowie Einrichtungen für Menschen mit Behinderung. Auch wenn das in der Leitlinie nicht reflektiert wird, kann dieser zehnte Sektor doch als ein Sonderfall gelten: Denn im Unterschied zu den anderen Sektoren geht es nicht um unverzichtbare Leistungen für die Gesellschaft (Energieversorgung, Lebensmittelproduktion, Krankenbehandlung etc.). Es geht nicht um die Systemrelevanz von Bildung und altersgemäßer Sozialisation, vielmehr werden Kitas und Schulen reduziert auf die Funktion, den Betrieb in den *anderen* Sektoren aufrechtzuerhalten – man könnte vielleicht von einer Systemrelevanz zweiter Ordnung sprechen. Es wird noch darauf zurückzukommen sein, dass diese impliziten Prämissen sinnvoll und plausibel sind, solange nach dem Modell eines Katastrophenfalls von zeitlich eng begrenzten Kita- und Schulschließungen ausgegangen wird, dass diese Degradierung aber weder politisch noch soziologisch verständlich gewesen wäre, wenn man geahnt hätte, dass die Sozialisation und Bildung aller Kinder von nicht-systemrelevanten Eltern über mehr als ein Jahr lang eingeschränkt werden sollte.

Vergleicht man das Konzept der Kritischen Infrastrukturen mit der Systemrelevanzsemantik der Finanzkrise, dann fällt auf, dass ersteres im öffentlichen Diskurs kaum eine Rolle spielt.^13^ Die KRITIS-Strategie fungiert also weniger als eine im politischen Raum strategisch eingesetzte Akteurskategorie, sondern eher als analytisches Instrumentarium, das eingebettet ist in Expertendiskurse der Verwaltung, der Sicherheitsbehörden und des Katastrophenschutzes. Im Bedarfsfall aber kann darauf sehr schnell zurückgegriffen werden, sodass es im März 2020 umstandslos in Systemrelevanzdiskurse eingebaut und damit öffentlichkeitswirksam werden konnte. Zusammenfassend könnte man hier von einer analytischen Kategorie sprechen, die bei Bedarf und mit Hilfe der Systemrelevanzsemantik in eine öffentlich verfügbare Akteurskategorie übersetzbar ist.

### Funktionalistische Differenzierungstheorien

Die dritte theoretische Perspektive, die hier genauer erläutert werden soll, ist oben mit dem Hinweis auf die Systemtheorie schon angeschnitten worden: die soziologische Theorie funktionaler Differenzierung. Diese hat ihre eigene Ideengeschichte, die keineswegs auf die Systemtheorie enggeführt werden darf (Tyrell 1998), die folgenden Überlegungen illustrieren die Problematik aber dennoch an den beiden Klassikern Talcott Parsons und Niklas Luhmann, deren Theorien in der hier interessierenden Hinsicht einen instruktiven Kontrast bilden.

Parsons war bekanntlich davon ausgegangen, dass jedes System – im Falle der Soziologie geht es ihm dabei um Handlungssysteme, doch die Idee dahinter ist abstrakter gebaut und lässt sich auch auf andere Systeme, etwa Organismen, beziehen – bestimmte Bestandsvoraussetzungen hat, die erfüllt sein müssen, damit das System funktioniert. Mit dem AGIL-Schema (Parsons 1966, 1971) identifizierte er dabei vier solcher unabdingbarer Funktionen: (1) „**A**daption“, (2) „**G**oal attainment“, (3) „**I**ntegration“ sowie (4) „**L**atent pattern maintenance“. Die gesellschaftlichen Institutionen, die diese Funktionen erfüllen – vulgärsoziologisch: die Wirtschaft, die Politik, die soziale Gemeinschaft und die Kultur – wären damit, auch wenn Parsons diesen Begriff nicht verwendet, „systemrelevant“. Denn wenn eine der vier Säulen wegbricht, kollabiert das System – d.h. die Gesellschaft – insgesamt. Luhmann wiederum grenzt sich von genau dieser Vorstellung dezidiert ab und erklärt die Idee des AGIL-Schemas für gescheitert bzw. für nur noch ideengeschichtlich interessant:^14^ „Die ältere soziologische Theorie hatte Funktionen als Bestandsvoraussetzung des Gesellschaftssystems definiert. Was damit gemeint war, ist unklar geblieben.“ (Luhmann 1997, S. 747)

Tatsächlich könnte die Haltung der beiden Theoretiker in dieser Frage unterschiedlicher nicht sein. Luhmanns Gesellschaftsbegriff ist grundlegend anders gebaut: Gesellschaft ist für ihn schlicht und einfach dasjenige umfassende soziale System, „das alle anderen sozialen Systeme in sich einschließt“ (Luhmann 1997, S. 78).^15^ Nach der kommunikationstheoretischen Wende fällt damit die Existenz der Gesellschaft mit der Existenz von Kommunikation zusammen: Solange kommuniziert wird, solange setzt sich Gesellschaft fort. In dieser Minimaldefinition hat die Idee, es könne für die Gesamtgesellschaft konstitutive Systeme geben, keinen Platz. Nur das Ende der Kommunikation schlechthin (etwa als Folge eines Weltuntergangs), nicht der Wegfall einzelner Systeme, könnte zum Ende der Gesellschaft führen. Anders als bei Parsons und vielen anderen soziologischen Theoretikern, die den Gesellschaftsbegriff weiter ausbuchstabieren und mehr oder weniger normativ aufladen, ist Gesellschaft bei Luhmann per definitionem nicht gefährdet. In gewisser Weise bestätigt die Geschichte diese Sichtweise, hat die Gesellschaft als Kommunikationszusammenhang doch unzählige Kriege, Pandemien und sonstige Katastrophen überlebt. Auf der anderen Seite könnte man an dieser Stelle den alten Streit fortsetzen, ob ein solcher Minimalbegriff von Gesellschaft für die Soziologie überhaupt einen Mehrwert hat oder ob man dann konsequenterweise nicht ganz auf den Gesellschaftsbegriff verzichten sollte (so dezidiert Schwinn 2001). Für diese Diskussion ist hier aber nicht der Raum, denn um die Relevanz der Systemtheorie für die Systemrelevanz-Problematik zu klären, muss die Frage anders formatiert werden: Sie lautet dann nicht mehr, ob und wie eine Gesellschaft große Katastrophen überlebt (sie tut es bzw. tat es bislang immer), sondern was es für ihre internen Strukturen bedeutet, wenn bestimmte Funktionssysteme ausfallen. In Anschluss an die oben zitierte Absage an Parsons’ Konzept der Bestandsvoraussetzungen schlägt Luhmann folgende Alternative vor:

Man kann nur induktiv vorgehen und mit einer Art Gedankenexperiment testen, wie das Gesellschaftssystem seine Strukturen zur Aufrechterhaltung seiner Autopoiesis ändern müßte, wenn bestimmte Funktionen nicht mehr erfüllt würden, – etwa Zukunftssicherung im Hinblick auf knappe Güter oder rechtliche Absicherung von Erwartungen oder kollektiv bindendes Entscheiden oder eine über selbstläufige Sozialisation hinausgehende Erziehung. Wir werden deshalb nicht von Bestandsvoraussetzungen sprechen, sondern von Bezugsproblemen, die auf die eine oder andere Weise behandelt werden müssen, soll die Gesellschaft ein bestimmtes Evolutionsniveau halten und auch andere Funktionen erfüllen können. (Luhmann 1997, 747)

Die Rede von „Bezugsproblemen“, die „auf die eine oder andere Weise“ behandelt werden können, verweist auf die funktionale Analyse, die, grob gesagt, nach funktionalen Äquivalenten sucht, anstatt bestimmte Systeme vorschnell als konstitutiv für das Ganze und damit als systemrelevant zu markieren. Das im Zitat aufgeworfene Beispiel einer Erziehung, die über selbstläufige Sozialisation hinausgeht, ist im Horizont der aktuellen Pandemie und der Debatte um die Bedeutung von Kitas und Schulen besonders naheliegend. Auch Luhmann, so darf man vermuten, ging davon aus, dass Kinder sozialisiert und erzogen werden müssen, und dass dafür in der funktional differenzierten Gesellschaft ein Erziehungssystem ausdifferenziert wurde, welches insbesondere vermittelt über die Institution der Schule dieses Bezugsproblem adressiert. Nur wäre es ein naturalistischer Fehlschluss, daraus zu folgern, dass Schulen systemrelevant für die Gesamtgesellschaft sind. Die funktionale Analyse fragt deshalb nach den funktionalen Äquivalenten, und das im März 2020 begonnene Realexperiment zeigt, dass die gesamte Sozialisation und schulische Bildung durchaus in die Familien und in die privaten Netzwerke verlagert werden kann.^16^ Ob das effizient ist oder nicht und welche längerfristigen Folgen das zeitigt, ist eine andere Frage; entscheidend ist zunächst nur, dass die Gesamtgesellschaft ganz gut weitermachen kann wie zuvor – sie kollabiert nicht nur nicht, sie ändert auch wenig an ihrer primären Differenzierungsstruktur. Ein etwas heikles Gedankenexperiment wäre nun, diese Frage für das Gesundheitssystem durchzuspielen, dessen Gefährdung in der Pandemiepolitik von Anfang an ein wichtiger Referenzpunkt war. Mit Luhmann müsste man hier konstatieren, dass auch im Falle einer durch Überlastung bedingten Schließung der Intensivstationen weder die funktionale Differenzierung noch die Versorgung der Kranken zusammenbrechen würde – diese würde nur nicht mehr in gleicher Weise in den bisher dafür zuständigen Organisationen stattfinden können, sondern beispielsweise in improvisierten Notorganisationen oder auch wieder in den Familien. Das wäre sicherlich weniger effizient, und diese Ineffizienz wäre durch die zu erwartende Zunahme von Todesfällen sichtbarer als im Falle von scheiternden Bildungskarrieren – aber in theoretischer Hinsicht sind das vergleichbare Fälle.

Während also Parsons von systemrelevanten Funktionen ausgeht, erklärt Luhmann, dass *nichts* unabdingbar systemrelevant ist in einer Welt der funktionalen Äquivalente. Zugleich liefert Luhmann, wenn auch eher nebenher, durchaus ein theoretisches Vokabular, mit dem Systemrelevanzdebatten kritisch beobachtet werden können. Entscheidend ist der letzte Satz im obigen Zitat: Es geht nicht darum, ob die Gesellschaft als solche zusammenbricht (sie tut es nicht), sondern darum, ob ein „bestimmtes Evolutionsniveau“ gehalten werden kann. Mit anderen Worten: Die Gesellschaft als solche überlebt sowohl einen Zusammenbruch des Gesundheitssystems wie eine Schließung der Schulen, aber wenn die evolutionär bewährten Einrichtungen zur Erfüllung der entsprechenden Bezugsprobleme längerfristig stillstehen, dann wird die zukünftige Gesellschaft eben nicht mehr auf dem erreichten „Evolutionsniveau“ operieren.

Zusammenfassend kann festgehalten werden, dass die differenzierungstheoretischen Perspektiven von Parsons und Luhmann einen rein analytischen Beitrag zur Systemrelevanz-Frage leisten. Weder das AGIL-Schema noch die funktionale Analyse können im öffentlichen und politischen Diskurs das leisten, was der Systemrelevanzsemantik gelingt; beide Konzepte lassen sich nur schwer in Akteurskategorien übersetzen. Darin kann für die Soziologie aber auch eine Chance liegen: ihr Beitrag könnte darin bestehen, das, was die Systemtheorie über Systemrelevanz weiß, in die öffentlichen Systemrelevanzdebatten einzubringen. Damit leistet sie, was Rosa mit Bezug auf Taylor, Giddens und Habermas als Kennzeichen der öffentlichen Soziologie betrachtet: „eine zur institutionellen Realität geronnene gesellschaftliche Selbstdeutung mit den begrifflichen Mitteln der Sozialwissenschaften noch einmal zu interpretieren“ (Rosa 2020, S. 206).

### Zwischenfazit

In Zeiten der Pandemie – und zukünftig möglicherweise auch in anderen gesellschaftlichen Krisen – provozieren alle drei skizzierten Theorieangebote ein Nachdenken darüber, welche gesellschaftlichen Bereiche und welche mit diesen Bereichen verknüpften Organisationen und Personen für die Gesellschaft unabdingbar sind und deshalb zwingend ohne Unterbrechung „in Betrieb“ sein müssen. Die drei Perspektiven sind eingebettet in drei Diskurszusammenhänge, die ein Denken in Kategorien gesellschaftlicher Differenzierung evozieren und dabei, oft eher beiläufig, eine Sortierung vornehmen zwischen mehr oder weniger relevanten sozialen Systemen. Dieses sortierende Denken operiert eher implizit als explizit, da meist nur die „relevanten“, nicht die „irrelevanten“ Bereiche beim Namen genannt werden. So wurde im „too big to fail“-Narrativ nur selten nachgefragt, ob es neben den Finanzinstitutionen auch andere unabdingbare Einrichtungen gibt, und in der KRITIS-Strategie wurde nicht festgelegt, dass Bildungs‑, Kultur- und Sporteinrichtungen unwichtig seien – sie blieben einfach unmarkiert. Dagegen neigen die soziologischen Differenzierungstheorien aus ihrer eigenen Logik heraus dazu, möglichst vollständige und gesättigte Listen mit gesellschaftlichen Bereichen zu erstellen und zugleich alle diese Bereiche für unabdingbar zu halten.^17^

Vor diesem Hintergrund ist es nicht nur eine praktisch-politische, sondern auch eine gesellschaftstheoretische Frage, ob es sich die moderne, funktional differenzierte und hochvernetzte Gesellschaft leisten kann, eines oder mehrere dieser Systeme für eine bestimmte Zeit auszuschalten oder einzufrieren. Nun hat sich auch in der Pandemie aus naheliegenden Gründen kaum ein Theoretiker so weit aus dem Fenster gelehnt, irrelevante Gesellschaftsbereiche zu benennen, um sie dann etwa der Politik zur Abwicklung vorzuschlagen. Zwischen den Zeilen aber ist eben dies ständig geschehen – wenn man etwa an den Fußball denkt (und damit indirekt an das System des Sports), der, wie oben erwähnt, im März 2020 zu einer Art Lehrbuchbeispiel wurde für Aktivitäten, auf die man nun erstmal verzichten müsse.

Im Horizont der provokativen Vorstellung, die Soziologie könne der Politik erklären, welche sozialen Systeme wichtig sind und welche nicht, springt ein am 7. April 2020 unter dem Titel „Simplifikation des Sozialen“ in der FAZ erschienener Beitrag von Rudolf Stichweh (2020) ins Auge. Dieser schließt analytisch an die im letzten Abschnitt diskutierte Differenzierungstheorie an, transformiert diese aber zugleich in eine radikale Form öffentlich-angewandter Gesellschaftstheorie. Aus heutiger Sicht erscheint dieser Beitrag als historisches Dokument bzw. als Dokumentation eines Theorieexperiments, welches in der dritten Woche des ersten Lockdowns und beeinflusst von der aufkeimenden Systemrelevanzdebatte eine Gesamtschau auf alle Funktionssysteme ausprobiert. Dieses Theorieexperiment, so kann man vermuten, fand krisenbedingt unter Zeitdruck und damit ungeschützt statt, erlaubte es dem Theoretiker aber eben deshalb, das zu tun, was die Soziologie sonst vermeidet: Im Ausnahmezustand zu spekulieren über die Systemrelevanz oder Irrelevanz jedes einzelnen Funktionssystems.^18^ Ausgangspunkt des Beitrags ist allerdings nicht die Frage nach irrelevanten Systemen, sondern vielmehr die Beobachtung, dass die Corona-Pandemie die funktional differenzierte Weltgesellschaft mit einer unbekannten Situation konfrontiert: die ansonsten eigenlogisch operierenden Funktionssysteme scheinen nun zumindest „zeitweilig“ einem einzigen Imperativ zu folgen, nämlich dem Überleben des Individuums. Stichweh argumentiert hier im Prinzip genauso wie später der oben schon zitierte Rosa, demzufolge „Lebensrelevanz“ plötzlich einen höheren Stellenwert gehabt habe als „Systemrelevanz“.

In dieser Situation, so Stichweh, spielt plötzlich das Gesundheitssystem die Hauptrolle. Dann kommen die Politik und die Wissenschaft, die Lösungen für das Problem finden müssen, und die Massenmedien, die darüber berichten, „wie die Verhaltensvorschriften aussehen“ und wie die Krise verläuft. Mit diesem Aufschlag begibt sich Stichweh auf das Terrain einer Ranglistenlogik und sortiert im Folgenden die Systeme nach ihrer abnehmenden Relevanz hinsichtlich des neuen Imperativs. Die Wirtschaft ist dann schon nur noch „punktuell systemrelevant“, das Erziehungssystem „wird eingestellt“ bzw. in die Zuständigkeit der Familien zurückverlagert, der Sport und das System der Kunst werden, sofern es um Anwesenheiten von Menschen geht, weitgehend „sistiert“ und das System der Religion erscheint als der „eigentliche Verlierer“, da es nicht benötigt wird zur sinnhaften Deutung der Krise. Eine Sonderrolle schließlich spielen in dieser Darstellung die Familie und Intimbeziehungen als ein Funktionssystem, „das niemand stillstellen und temporär auflösen möchte“, das aber zugleich als „unrealistisch stabil gedacht“ werde. Die Prämisse, dass dieses Funktionssystem in der Krise die Leistungen der anderen Systeme übernimmt, könnte, so Stichweh, schon „nach wenigen Wochen immer problematischer werden“. Mit anderen Worten: Nach drei Wochen Lockdown konnte sich der Gesellschaftstheoretiker nicht vorstellen, dass dieses Abwälzen länger als wenige Wochen durchzuhalten wäre – wie wir heute wissen, waren es am Ende für viele Eltern und Schüler mehr als 30 Wochen.^19^ Im Fazit des vorliegenden Textes wird nochmals deutlich werden, wie wichtig dieser indirekte Hinweis auf die Bedeutung der Zeitdimension ist.

Nun geht es an dieser Stelle nicht darum, diesen faszinierend ungeschminkten Entwurf soziologisch weiter auszubuchstabieren. Festgehalten werden soll nur, dass hier mehr oder weniger nebenbei ein Aspekt explizit gemacht wird, der bei vielen vorsichtiger argumentierenden soziologischen Essays und Feuilleton-Beiträgen aus der frühen Phase der Pandemie nur angedeutet wurde: dass man nämlich im Blick auf die Vielfalt der gesellschaftlichen Teilbereiche eine Linie ziehen könnte – oder dass die Politik in Notlagen gezwungen sein könnte, eine Linie zu ziehen – zwischen systemrelevanten Bereichen (Politik, Wirtschaft, Gesundheit, Wissenschaft usw.) und systemirrelevanten Bereichen (Kunst, Sport, Religion, Erziehung usw.). So gut die Gründe sind, sich gegen jede derartige ins binäre kippende Logik der Sortierung zu wehren, so wenig lässt sich ignorieren, dass diese Leitunterscheidung den Pandemiediskurs, die Pandemiepolitik und auch die soziologische Beobachtung dieser Politik mitgestaltet hat. Als latente Struktur, als Beobachtungsschema, das über längere Zeit in der Politik, in der Wissenschaft, in den Medien und in der Alltagskommunikation zum Einsatz kam und kommt, hat diese Unterscheidung – auch wenn sie als analytisches Beobachtungsschema kaum jemanden befriedigen dürfte – gute Chancen, sich evolutionär weiterzuentwickeln und über die Pandemie hinaus eine Rolle zu spielen in der gesellschaftspolitischen Semantik des 21. Jahrhunderts.

Die öffentliche Soziologie wiederum sollte regelmäßig reflektieren, ob und wie ihre analytischen Kategorien einerseits durch Akteurskategorien aus der Praxis beeinflusst sind und andererseits umgekehrt dazu beitragen können, die in der Politik und im öffentlichen Raum strategisch eingesetzten Akteurskategorien kritisch zu hinterfragen sowie konstruktiv weiterzuentwickeln. Der schillernde Begriff der Systemrelevanz ist nur ein Beispiel für die fortwährende Äquilibration von sozialwissenschaftlichem und öffentlichem Diskurs.

## Die Zeitdimension als Leerstelle des Pandemiediskurses

Die Debatten darüber, welche Bereiche systemrelevant sind und welche nicht, welche zuerst und welche zuletzt geschlossen werden sollten, werden uns noch lange in Erinnerung bleiben. Der Pandemiediskurs, so kann man die Zeit vom ersten Lockdown im März 2020 bis zur „Bundesnotbremse“ im April 2021 zusammenfassen, hatte sich in einen zweipoligen Argumentationsfeld verfangen: Je nach Gesamtbilanz der Öffnungen und Schließungen schwang das Pendel in Richtung Lockdown oder in Richtung Lockerungen. Geburtstagsfeiern, Hochzeiten, Friseursalons, Fußballspiele, Konzerte, Biergärten, Restaurants, Kitas und Schulen wurden zu Variablen in dieser imaginierten Gleichung. Bei der damit im kollektiven Denken verankerten Frage – „Was können wir zumachen, um Kontakte zu reduzieren?“ – gab es allerdings erstaunlicherweise einen Aspekt, der kaum thematisiert wurde: den Zeithorizont. Die Frage lautete, was warum zugemacht werden muss, aber kaum: wie lange? Und wenn doch, wurde gerne ausgewichen, beschwichtigt („drei Tage früher in die Weihnachtsferien“)^20^ oder es wurde auf die in den jeweiligen Beschlüssen genannte Datierung der geltenden Maßnahmen verwiesen, ergänzt mit dem Hinweis, dass man, wenn der Zeitpunkt gekommen sei, die Infektionszahlen anschauen und weitersehen müsse. Die eigentliche, wenn auch mehr oder weniger verschleierte Antwort war meistens ein „bis auf weiteres“. Wenn ein Ende versprochen wurde, dann nicht im Sinne eines ablaufenden Zeitraums („der Lockdown wird nicht länger als vier Wochen dauern“), sondern geknüpft an Erfolgsbedingungen („bis die Inzidenzen unter 50 oder unter 35 oder am besten noch tiefer gesunken sind“).

Natürlich geht es hier nicht darum, der Politik oder bestimmten Akteuren dieses Hinhalten oder Verschweigen der tatsächlich zu erwartenden Zeithorizonte vorzuwerfen. Vielmehr geht es darum, einen Diskurseffekt zu verstehen: Diese Leerstelle, dieses Nicht-Thematisieren der Zeitdimension ist ein konstitutives Merkmal des Pandemiediskurses. Zugleich kann und muss man aus sozialwissenschaftlicher Sicht darauf hinweisen, dass wir über Systemrelevanz nicht sinnvoll sprechen können, ohne zugleich zu reflektieren, *wie lange* die Gesellschaft bestimmte Institutionen auf Eis legen kann, ohne dass diese nachhaltig Schaden nehmen. Ein paar Gedankenexperimente genügen, um zu illustrieren, wie unabdingbar Systeme jeder Art in der Zeit operieren und nicht ohne eine jeweils eigene Dauer zu denken sind: Der Mensch als organisches System kann gut ein paar Stunden nichts trinken, wenn er aber, je nach sonstigen Umständen, zwei bis sechs Tage nichts trinkt, ist er tot. Ebenso, wenn er, wiederum je nach sonstigen Umständen, 30 Tage bis ein Jahr lang nichts isst; obwohl umgekehrt das Fasten über einen gewissen Zeitraum sogar gesund sein kann. Ein anderes und in der Technikfolgenabschätzung gut erforschtes Beispiel führt unmittelbar in die oben diskutierte Problematik Kritischer Infrastrukturen. Die Notstromversorgung in Krankenhäusern ist gängigerweise darauf ausgelegt, die essenziellen Systeme im Notfall für mindestens 24 Stunden mit Strom zu versorgen; schon nach acht Stunden allerdings ist von einer deutlichen Beeinträchtigung der medizinischen Versorgung auszugehen (Frädrich 2012). Schätzungen gehen davon aus, dass bei einem längeren Blackout das Krankenhaus den Betrieb schon nach ein bis zwei Tagen nicht mehr aufrechterhalten kann und evakuiert werden muss (Petermann et al. 2010). Ein drittes Beispiel führt die Bedeutung solcher Zeithorizonte noch deutlicher vor Augen. Am Anfang der Atomkatastrophe von Tschernobyl stand ein Test, mit dem herausgefunden werden sollte, ob bei einem Stromausfall die Energie der in Bewegung befindlichen Turbinen ausreicht, die kritischen 40 bis 50 Sekunden zu überbrücken, bevor die Notstromdieselaggregate hochgefahren sind und hinreichend Strom produzieren, um die Kühlung des Reaktors aufrechtzuerhalten (vgl. Informationskreis Kernenergie 1996). Auch wenn Tschernobyl letztlich aus einem anderen Grund kollabiert war, zeigt auch dieses Beispiel, dass sozio-technische Systeme über eine zeitliche Reserve verfügen, eine berechenbare Dauer, über die sie stillgestellt oder von der Versorgung mit Ressourcen abgeschnitten werden können. Sobald aber diese überbrückbare Zeit überschritten wird – man könnte hier von einer Lockdown-Eigenzeit sprechen – kollabiert das System.

Die Übertragung dieser Eigenzeit-Problematik auf unterschiedlich komplexe soziale Systeme oder gesellschaftliche Bereiche – Friseurbetriebe, Schulen, Restaurants oder, auf der nächsthöheren Ebene, das Erziehungssystem oder „die Kultur“ – ist nicht unproblematisch, und die Gedankenexperimente werden hierbei notwendig spekulativer. Die soziologische Frage aber bleibt: Wie lange können bestimmte Praktiken, Tätigkeiten, Organisationen oder Funktionssysteme in einen Pausenbetrieb wechseln, ohne Schaden zu nehmen? Zunächst sind die Zeithorizonte hier ganz andere als bei sozio-technischen Systemen; es geht nicht um Sekunden, Stunden oder Tage, sondern eher um Monate oder Jahre; außerdem sind dies keine Zeithorizonte mehr, die sich wissenschaftlich berechnen lassen – die Berechnung der Lockdown-Eigenzeiten von sozialen Systemen wäre ein Beispiel für eine Kategorie von Fragen, die Alwin Weinberg (1972) als „transwissenschaftlich“ bezeichnet. Aber erneut können wir mit Hilfe von Gedankenexperimenten zumindest darüber nachdenken, ob in sozialen Zusammenhängen nach einer gewissen Zeit des Stillstands Strukturveränderungen stattfinden, die dazu führen, dass das System nach dem Lockdown nicht mehr gleichermaßen leistungsfähig ist wie vor dem Lockdown.

Hinweise dazu liefert beispielsweise die Glücksforschung, die unter anderem untersucht, wie sich bestimmte Ereignisse im Lebensverlauf auf die subjektiv empfundene Lebensqualität von Menschen auswirken. Die so genannte Adaptionsthese etwa besagt, dass sowohl sehr positive wie auch sehr negative Ereignisse nach einer bestimmten Zeit kompensiert und damit gewissermaßen vergessen werden – das subjektive Wohlbefinden pendelt dann zurück zur „baseline“ (Headey und Wearing 1989; Diener et al. 1999). Ein Schicksalsschlag etwa kann bedeuten, dass ein Mensch für ein Jahr am Boden zerstört ist, nach ein oder zwei Jahren aber hinsichtlich seiner Lebensqualität wieder ungefähr da steht, wo er vor diesem Schicksalsschlag stand. Man könnte argumentieren, dass die im Medium des Lockdowns operierende Pandemiepolitik letztlich auf diese Karte gesetzt hat; sie hoffte, dass die Resilienz der Menschen und der sozialen Systeme ausreicht, um nach der Pandemie und trotz harter Einschnitte dieselbe Lebensqualität zu erreichen wie vor der Pandemie. Doch die Glücksforschung verweist auf der anderen Seite auch auf Ereignisse wie Armut oder Langzeitarbeitslosigkeit, die *nicht* ohne Langzeitfolgen bleiben – selbst wenn sich die Arbeitssituation wieder ändert (Böhnke 2009; Stöbel-Richter et al. 2012). Während etwa eine kürzere Arbeitslosigkeit im Allgemeinen gut weggesteckt wird, zeitigt eine Langzeitarbeitslosigkeit andere Effekte; die Betroffenen kommen, statistisch betrachtet, nicht mehr auf die gleiche Lebensqualität und nicht mehr auf die gleiche Arbeitsleistung. Lange, unfreiwillige Arbeitslosigkeit, die eine bestimmte Schwelle überschreitet, kann als eine Art individueller Lockdown konzeptualisiert werden – und das wäre ein Hinweis darauf, dass die Pandemiepolitik nicht zu sehr auf die Adaptionsthese vertrauen sollte. Auch hier zeigt sich wieder, dass ein komplexes Phänomen wie „Arbeitslosigkeit“ aus sozialwissenschaftlicher Perspektive nicht dasselbe bleibt, wenn es kürzer oder länger dauert. So wie ein Stromausfall von zwölf Stunden in einem Krankenhaus etwas substanziell anderes bedeutet als ein Stromausfall von 48 Stunden, so ist eine Arbeitslosenzeit von sechs Monaten nicht ohne Weiteres mit einer von 24 Monaten vergleichbar. Die Differenz ist in beiden Fällen nicht einfach ein Faktor 4, sondern ein qualitativer Unterschied hinsichtlich der schwer berechenbaren, unter Umständen aber desaströsen Effekte.

Was folgt daraus im Blick auf die schwierige Unterscheidung von systemrelevanten und nicht-systemrelevanten Gesellschaftsbereichen? Im Prinzip ist die Soziologie aufgefordert, für alle im Pandemiediskurs als „nicht systemrelevant“ markierten Entitäten genauer zu eruieren und empirisch zu erforschen, was die Effekte einer Schließung über die Zeit sind. Was bedeutet es für Sportvereine, Hobbyfußballer, Friseursalons oder Berufsmusikerinnen, wenn sie ihre jeweilige soziale Praxis über Monate oder Jahre gar nicht, nur eingeschränkt oder nur in substanziell veränderter Form (etwa virtuell) aufrechterhalten können? Diese Frage kann nicht pauschal, sondern müsste für jeden gesellschaftlichen Teilbereich und für jedes soziale System genauer ausbuchstabiert werden. Auch die Gesamtgesellschaft muss hier im Auge behalten werden. So weist Klaus Dörre zurecht darauf hin, dass das „Social Distancing“, wenn es „lange“ andauert, auf eine „radikale Entgesellschaftung und Entgemeinschaftung“ hinauslaufen könnte (Dörre 2020, S. 182). Auch hier aber bleibt – wie in allen transwissenschaftlichen Fragen – völlig offen, wann „lange“ zu lange ist.

Anstelle eines Fazits und im Sinne eines Ausblicks auf mögliche Forschungen schließt der vorliegende Text mit einer These zur Zukunft des von der Pandemiepolitik besonders betroffenen Erziehungssystems. Es ist nicht auszuschließen, dass das Realexperiment der in Deutschland insgesamt ca. 38 Wochen^21^ dauernden (partiellen) Kita- und Schulschließungen zu „Systemschäden“ geführt hat, sodass nachträglich das Bewusstsein um die „Systemrelevanz“ der Kitas und Schulen oder, abstrakter, das Wissen um die Bedeutung einer „über selbstläufige Sozialisation hinausgehende[n] Erziehung“ (Luhmann) steigen könnte. Möglicherweise werden wir in einigen Jahren, nach der international vergleichenden, soziologischen, ungleichheitstheoretischen und erziehungswissenschaftlichen Aufarbeitung dieses Realexperiments, über einen erweiterten Begriff von Kritischen Infrastrukturen verfügen, in den die Erkenntnis eingeflossen ist, dass auch in Zeiten der Pandemie manche Institutionen nicht über eine gewisse Dauer hinweg geschlossen werden können. Vielleicht kommt es aber auch ganz anders und die These wird durch die Entwicklung der nächsten Jahre widerlegt, sodass man sich zukünftig vielleicht noch stärker als vor der Pandemie auf die Systemrelevanz der harten Funktionssysteme, auf Politik, Wirtschaft, Gesundheit konzentrieren wird – wohl wissend, dass andere Bereiche auch mal für ein paar Monate oder ein Jahr pausiert oder privatisiert werden können. Die Soziologie wiederum entscheidet schon in der näheren Zukunft, ob sie die Herausforderung annimmt, durch eigene empirische Forschung zur Beantwortung dieser teilweise stark politisierten Fragen beizutragen, oder ob sie sich, dem in der Pandemie eintrainierten Verantwortungsgefühl entsprechend, auf die intellektuelle Unterfütterung und Kommentierung dessen beschränkt, was gesellschaftspolitisch Realität geworden ist.

## Footnotes

1 Für eine Diskussion des methodischen Potenzials sowie der methodischen Grenzen des Google Ngram Viewer siehe Chumtong und Kaldewey (2017).

2 Aktualisierte Datenerhebung am 05.01.2022. Zu beachten ist, dass es sich sowohl bei Google Scholar wie beim DIP um laufend aktualisierte Datenbanken handelt, d.h. spätere Abfragen können zu anderen Zahlen führen.

3 Es sind im betrachteten Korpus und Zeitraum nur Männer, deshalb wird hier bewusst nicht gegendert. Grundsätzlich versucht der vorliegende Essay, weibliche und männliche Formen zufällig zu wählen. Sofern in den zitierten Quellen (etwa in der *FAZ*) das generische Maskulinum verwendet wird, wird dieses im Text beibehalten (auch ohne explizite Kennzeichnung als Zitat).

4 Eine solche explizite Bezeichnung der Schulen und Kitas als *nicht* systemrelevant ist bis heute wenig gebräuchlich; umgekehrt hatte zunächst auch niemand mit Nachdruck Systemrelevanz behauptet. In der binären Logik des in Gang gesetzten Diskurses bedeutet das, dass keine Systemrelevanz vorliegt. Trotz hörbarer Sorgen von (weiten Teilen) der Elternschaft, Kinderärzten und den Kultusministerien war es dann bis Mitte 2021 nicht mehr gelungen, die Schulen gegen die Eigenlogik des Diskurses als „hinreichend“ systemrelevant darzustellen, um sie offen zu halten.

5 In Luhmanns Schriften etwa taucht der Ausdruck zwar vereinzelt auf, doch letztlich bleibt es dabei bei einer alltagssprachlichen Bedeutung: etwas (z.B. ein bestimmter Mechanismus) ist für etwas (ein bestimmtes System) relevant. Im Endeffekt ist die Theoriestelle, die der Systemrelevanzbegriff einnehmen könnte, in der Systemtheorie durch den Begriff der Funktion bereits besetzt. Das zeigt beispielhaft das folgende Zitat (eine der wenigen Stellen, bei denen Luhmann den Systemrelevanzbegriff verwendet): „Diese Schwierigkeiten beruhen auf der Grundkonzeption, daß Funktionen und Dysfunktionen besondere Arten von Wirkungen sind, die logisch gleichwertig nebeneinanderstehen und sich nur durch ihre umgekehrte Systemrelevanz unterscheiden.“ (Luhmann 2005, S. 27).

6 Eine weniger optimistische Skizze der Bedeutungsverschiebung von Systemrelevanz findet sich bei Simon Scharf (2020). Zunächst, so Scharf im Blick auf die Finanzkrise 2007–2009, handelt es sich bei Systemrelevanz um eine „vorrangig ökonomische Kategorie“. Durch eine inflationäre Bedeutungsextension im Zuge der Coronakrise verkomme dieser dann zu einem „unscharfen Kampfbegriff“ (ebd., S. 189), der ökonomische Denkweisen in Debatten hineinträgt, in die sie nicht hineingehören. Am Ende führe das zu einem „Aufkündigen der Solidarität in Form einer starken Konkurrenzlogik im Ringen um die eigene Relevanz im ‚System‘“ (ebd., S. 192).

7 Etabliert wurde etwa das Konzept von „Systemically Important Financial Institutions“ (SIFIs). Hieran wiederum anschließend legt das 2009 eingerichtete „Financial Stability Board“ (FSB) jährlich eine Liste mit „Global Systemically Important Banks“ (G-SIB) und „Global Systemically Important Insurers (G-SII) vor.

8 Dass die offizielle Etablierung des Konzepts Kritischer Infrastrukturen im Jahr 2009 so unmittelbar auf die Finanzkrise folgt, ist vermutlich ein Zufall. Ob und inwieweit die Finanzkrise Einfluss auf die Ausarbeitung und Implementierung der KRITIS-Strategie hatte, kann hier nicht geklärt werden.

9 Die Darstellung bezieht sich auf die Websites von BBK und BSI (Stand Mai 2021). Im Juli 2021 wurde im Zuge der Novellierung des BSI-Gesetzes als weiterer Sektor die „Siedlungsabfallentsorgung“ ergänzt. Im Originaldokument (BMI 2009, S. 5) ist die Kategorisierung etwas anders gebaut: Zwar sind die neun Sektoren dort schon angelegt, es wird aber zusätzlich unterschieden zwischen „technischen Basisinfrastrukturen“ (Energie, Informations- und Kommunikationstechnologie, Transport und Verkehr, Wasser) sowie „sozioökonomischen Dienstleistungsinfrastrukturen“ (Gesundheit, Ernährung, Notfall- und Rettungswesen, Katastrophenschutz, Parlament, Regierung, öffentliche Verwaltung, Justizeinrichtungen, Finanz- und Versicherungswesen, Medien und Kulturgüter).

10 Aus soziologischer Sicht kann man das so interpretieren, dass im BSI-Gesetz die Sektoren stärker als soziotechnische Systeme und weniger als Systeme im Sinne klassischer Differenzierungstheorien konzipiert sind.

11 Die letzte Übersicht ist datiert auf den 5. Februar 2021, bezieht sich also schon auf den zweiten Lockdown. Siehe https://www.kritis.bund.de/SharedDocs/Downloads/BBK/DE/Sonstiges/Covid-19_Kritis_Schulen_Kitas.html (zuletzt aufgerufen am 18.08.2021). Für die erste Fassung, siehe https://www.bdsw.de/images/aktuell/Kritische_Infrastruktur.pdf (zuletzt aufgerufen am 01.02.2022).

12 Empirische Studien, wie häufig solche aus der Perspektive der Geschlechtergerechtigkeit durchaus interessanten Konstellationen waren, liegen m.W. nicht vor. Untersucht sind allerdings die Verschiebungen in der Aufteilung der Care-Arbeit als Folge der Pandemie. Für einen Überblick siehe Krohn (2020).

13 Aktuell – diese Zeilen sind Anfang Januar 2022 ergänzt worden – zeichnet sich eine Änderung ab, da der von der neuen Bundesregierung im Dezember 2021 eingesetzte Corona-Expertenrat bereits in seiner ersten Stellungnahme und mit Blick auf Entwicklungen in Großbritannien betont hat, dass ein besonderes Risiko der Omikron-Welle darin liegen könnte, dass hohe Krankenstände zum Ausfall Kritischer Infrastrukturen führen.

14 Luhmann selbst unterschätzt aber möglicherweise, wie sehr dieses Viererschema implizit viele soziologische und historische Theorien und möglicherweise auch unser Alltagswissen über die Gesellschaft geprägt hat (siehe Kaldewey 2015, S. 94 ff.).

15 In einer legendären Minimaldefinition im Lexikon zur Soziologie (hrsg. v. Werner Fuch-Heinritz et al.) lautet Luhmanns Definition für Gesellschaft: „das jeweils umfassendste System menschlichen Zusammenlebens. Über weitere einschränkende Merkmale besteht kein Einverständnis.“ (zitiert aus der 3. Auflage 1994).

16 An dieser Stelle wird häufig das Gegenargument vorgebracht, dass der kommunikative Bezug zur Schule nicht gekappt, sondern, wenn auch in der Praxis mehr schlecht als recht, digitalisiert wurde. Die zweifellos wichtige Frage, ob und inwieweit die Digitalisierung das Pausieren gesellschaftlicher Bereiche im Lockdown zu mildern oder gar zu kompensieren vermag, würde den Rahmen des vorliegenden Textes allerdings sprengen. Erlaubt sei aber der Hinweis, dass inzwischen einige Studien vorliegen, die insbesondere für den ersten Lockdown zeigen, dass die Lernerfolge bei vielen Schülerinnen und Schülern (insbesondere aus nicht privilegierten Familien) während des ersten Lockdowns quasi stagnierten oder es sogar zu Kompetenzeinbußen kam. Distanzunterricht, so das Resultat der Metastudie von Hammerstein et al. (2021), erwies sich als in etwa so effektiv wie Sommerferien.

17 Interessanterweise produzieren verschiedene klassische Soziologen (und vereinzelte Soziologinnen) aus unterschiedlichen Schulen seit langem erstaunlich ähnliche Listen, obwohl sie sich ansonsten in keinerlei Weise einig sind, wie man die gelisteten Systeme oder Bereiche genau definiert oder auch nur benennt („Wertsphären“, „Teilsysteme“, „soziale Felder“, „Funktionssysteme“ etc.). Im systemtheoretischen Kontext wurde vor diesem Hintergrund eine – halb-ernst oder halb-ironisch gemeinte – Kanonisierung der Liste von Funktionssystemen vorgeschlagen (Roth und Schütz 2015).

18 Genaugenommen geht Stichweh in dem Beitrag zehn Systeme durch; auffallend bei seiner Liste ist das Fehlen des Rechtssystems.

19 Siehe dazu ausführlicher die Anmerkungen zu den Schulschließungen im nächsten Abschnitt (insbes. Fn. 21).

20 Wörtlich sagte Bundeskanzlerin Angela Merkel in ihrer viel beachteten emotionalen Ansprache im Bundestag am 9. Dezember 2020: „Was wird man denn im Rückblick auf ein Jahrhundertereignis mal sagen, wenn wir nicht in der Lage waren, für diese drei Tage noch irgendeine Lösung zu finden?“.

21 Diese Dauer der Kita- und Schulschließungen lässt sich nicht eindeutig benennen, zum einen weil Zahlen dazu nicht zentral erfasst sind und sich in den einzelnen Bundesländern unterscheiden, zum anderen weil die Unterscheidung von vollständigen und partiellen Schließungen nicht leicht operationalisierbar ist. Für die hier angegebene Schätzung von 38 Wochen wurde auf Daten des European Centre for Disease Prevention and Control (ECDC) zurückgegriffen (Data on country response measures to COVID-19, Stand Sept. 2021). Die 38 Wochen beinhalten Phasen reiner Schließung bzw. reinen Distanzunterrichts sowie von Teilschließungen bzw. Wechselunterricht. In diese Zeiten fallen ca. 6 Wochen reguläre Schulferien. Daraus folgt, dass während 32 Wochen kein regulärer Unterricht stattgefunden hat, was ca. 80 % eines regulären Schuljahres (mit 40 Wochen Unterricht) entspricht. Im Vergleich mit anderen europäischen Staaten, das zeigen die Daten des ECDC, steht Deutschland damit in der „Spitzengruppe“. Siehe dazu auch die instruktive Studie von Freundl et al. (2021), die mit anderer Berechnungsgrundlage zu einem ähnlichem Resultat kommt (183 Schließtage) und diese Zahlen mit anderen europäischen Ländern vergleicht.
